# Saikosaponin A Inhibits Breast Cancer by Regulating Th1/Th2 Balance

**DOI:** 10.3389/fphar.2019.00624

**Published:** 2019-06-04

**Authors:** Xin Zhao, Jinyu Liu, Shasha Ge, Chen Chen, Shuang Li, Xiaoyu Wu, Xuanye Feng, Yueqi Wang, Dayong Cai

**Affiliations:** ^1^Institute of Medicinal Plant Development, Chinese Academy of Medical Sciences, Peking Union Medical College, Beijing, China; ^2^School of Pre-clinical Medicine, Beijing University of Chinese Medicine, Beijing, China

**Keywords:** Saikosaponin A, breast cancer, anti-tumor immunity, T lymphocytes, Th1/Th2 balance

## Abstract

Saikosaponin A (SSa) is isolated from the dried root of *Radix Bupleuri*, an herb widely used in traditional Chinese medicine, exerting antitumor activities. The T helper cell type 1(Th1)/Th2 balance is associated with antitumor immunity in breast cancer. The present study aimed to investigate the effects of SSa on Th1/Th2 balance in breast cancer and to explore the underlying mechanisms. Breast cancer in rats was induced by intragastrical administration of 7,12-dimethyl-benz[a] anthracene once (100 mg/kg). At d_91_, the rats suffering from tumors were randomly divided into three groups and treated with vehicle solution (control group), tamoxifen (TAM group), and SSa (SSa group) daily for 56 days, respectively. The tumor volume reduction ratio and tumor cell proliferation were detected to assess the antitumor effect of SSa. The positive staining numbers of CD8+ and CD4+ T cells infiltrated in breast tumors were measured by immunohistochemistry to evaluate the antitumor immunity of SSa. Cytokine levels in serum secreted by Th1 cells [interferon gamma (IFN-γ), interleukin (IL)-12] and Th2 cells (IL-4, IL-10) were detected to evaluate Th1/Th2 balance. The related molecules of IL-12/signal transducers and activators of transcription 4 (STAT4) pathway were detected by immunohistochemistry staining, RT-PCR, and Western blot to explore the mechanisms of SSa. The results showed that, compared with the control group, SSa significantly inhibited tumor growth and tumor cell proliferation. SSa enhanced antitumor immunity, which was demonstrated as increased CD8+ T cells and CD4+ T cells infiltrated in tumors. SSa shifted Th1/Th2 balance toward Th1, which was confirmed as increased serum IFN-γ and IL-12 levels, while decreased serum IL-4 and IL-10 levels. SSa increased IL-12, IL-12 receptor, and phosphorylated STAT4 expressions to promote Th1 differentiation. In conclusion, the present work suggested that SSa could inhibit breast cancer growth by shifting Th1/Th2 balance toward Th1. The underlying mechanism may involve activation of the IL-12/STAT4 pathway that induced Th1 differentiation.

## Background

Breast cancer is the most common malignant tumor with leading cancer-associated mortality among women (Chen et al., 2016; Siegel et al., 2018). The host immune system plays an important role in the development and metastasis of breast cancer (Gil Del Alcazar et al., 2017). Targeting antitumor immunity has been considered as a promising therapeutic strategy for treating breast cancer (Hammerl et al., 2017).

In breast cancer, the tumor-infiltrating lymphocytes (TILs) have been recognized as a biomarker of antitumor response. Among the different TIL subsets, CD4+ or CD8+ T lymphocytes can recognize tumor antigens or eliminate tumor cells. CD8+ cytotoxic T cells mediate tumor-specific adaptive immunity that attacks tumor cells. CD4 + cells have two distinct subsets of T helper (Th)1 and Th2 cells with different cytokine production and underlying mechanism of action in breast cancer (Dushyanthen et al., 2015). Th1 cells produce tumor necrosis factor alpha, interferon gamma (IFN-γ), interleukin (IL)-2, and IL-12, which mediate antitumor effects. The Th2 cells produce IL-4 and IL-10 and contribute to favor tumor growth by inhibiting the host immune system. IL-12 promotes the differentiation of Th1 cells, whereas IL-4 promotes the development of Th2 cells. Signal transducer and activator of transcription 4 (STAT4) is activated upon IL-12 binding to the receptor and then induces naive CD4+ T cells differentiating into Th1 cells. In addition, the Th1 cells produce IFN-γ (Athie-Morales et al., 2004). The clinical data indicate that Th1/Th2 imbalance had been observed with an elevation of Th2-released cytokines in breast cancer patients. Further, patients with Th1 dominant response show higher survival and lower cancer reoccurrence rate. Therefore, development of new strategies that regulate Th1/Th2 balance may be beneficial for breast cancer treatment.

Saikosaponin A (SSa) is a triterpenoid glycoside extracted from *Bupleurum Radix*, exhibiting anti-inflammatory, immunity-regulatory, and anticancer activity. SSa induces apoptosis of human breast cancer MDA-MB-231 and MCF-7 cells (Chen et al., 2003). However, the effects of SSa on antitumor immunity or TILs in breast cancer are not clear.

Therefore, in this study, we investigated whether SSa could regulate Th1/Th2 balance. The antitumor effects and underlying mechanisms of SSa against breast cancer were explored.

## Materials and Methods

### Animals and Regents

Fifty female SD rats were obtained from the Animal Centre of the Chinese Academy of Medical Sciences. All animals were kept under a 12-°h light/dark cycle and temperature (25.0 ± 0.2°C)- and humidity (45 ± 2%)-controlled specific pathogen-free environment, fed standard rodent pellets, and allowed free access to filtered water. All experimental procedures were approved by the Ethics Committee of the Institute of Medicinal Plant Development, CAMS & PUMC.

The 7,12-dimethyl-benz [a]anthracene (DMBA) was purchased from Sigma. Tamoxifen (TAM) was purchased from Shanghai Forward Pharmaceutical Co. Ltd. SSa (PubChem CID: 45358148, purity: 70.5% ± 0.02%) was purchased from Shanghai Jimian Shiye Co. Ltd.

### Experimental Design

Breast cancer was induced by intragastrical administration with a single dose of DMBA (100 mg/kg) once. At d_91_, 36 rats suffering from tumors were selected and randomly divided into three groups (*n* = 12 in each group): control group, TAM group, and SSa group. The rats in each group were intragastrically administrated with 0.1% carboxymethylcellulose sodium (CMC, 10 mL/kg), TAM (5.6 mg/kg), or SSa (35 mg/kg) daily for 56 days, respectively. The length (*a*) and width (*b*) of tumors were measured every week. Tumor sizes were calculated using the following formula: tumor volume (TV, cm^3^) = 1/6πab^2^. The tumor volume reduction rate (IR) in the therapy group was calculated using the formula: (TV_control group_ − TV_therapy group_)/TV_control group_ × 100%. At d_147_, 2 h after final administration, rats were anesthetized by 10% chloral hydrate, then blood was obtained from aorta abdominals. The tumors were removed, weighed, and then dissected into three parts and stored for assays, respectively.

### Cytokine Levels in Serum

The blood samples were centrifuged at 3,000×*g* for 15 min at 4°C. Then, serum was collected and stored at −80°C until the assays. Levels of IFN-γ, IL-12, IL-4, and IL-10 were detected by the enzyme-linked immunosorbent assay kit (Boster, Wuhan, China). The values of optical density were determined at 450 nm, and the concentrations were calculated by converting the optical density against a standard curve.

### Immunohistochemistry and Morphometry

Tumor tissues were excised and fixed in 10% formalin (phosphate-buffered saline) for 24 h. Following paraffin embedding, the tissue sections were performed with immunohistochemistry (IHC) staining using standard protocols. Briefly, sections were incubated with primary antibodies against Ki-67 (1:200, Boster, Wuhan, China), CD8 (1:200, Santa Cruz, CA, USA), CD4 (1:200, Santa Cruz, CA, USA), IL-12 (1:400, Santa Cruz, CA, USA), IL-12 receptor (IL-12R, 1:400, Santa Cruz, CA, USA), or pSTAT4 (1:200, Santa Cruz, CA, USA), respectively. Then, an avidin–biotin peroxidase kit was used according to the manufacturer’s instructions (Boster, Wuhan, China). One set with hematoxylin counterstaining was used for cellular location. The other set without counterstaining was used for morphometry. All of the images were acquired by a Digital Pathology system (Kfbio, Ningbo, China) under 40× magnifications. The five images were randomly acquired from one slide.

The Ki-67 expression was calculated as percentages of positive stained tumor cell nuclei from the total number of tumor cells with at least 500 tumor cells for each tumor. The expression of CD4 and CD8 was quantified as the number of positive cells counted in each image. The mean optical density (OD), positive area (AP), and observed area (AT) of IL-12, IL-12R, and pSTAT4 protein IHC expressions had been analyzed with Image Pro-Plus 7.0.1 software and calculated by the formula [OD × (AP ÷ AT)^3/2^]. The average of five random fields generated a single data for statistic analysis.

### Quantitative Reverse Transcription Polymerase Chain Reaction Analysis

Total RNA was extracted from the tumor tissues using TRIzol according to the manufacturer’s instructions (Promega). Trace contamination of DNA was removed by DNase digestion (Promega). The 2.0 μg of total RNA was reversely transcribed using SuperScript III Reverse Transcriptase (Invitrogen). The real-time quantitative PCR was performed in IQ 5.0 ABI 7500 (Bio-Rad, USA) using TaKaRa SYBR Premix Ex Taq™. The sequences of primers were as follows: IL-12, 5′-GACAAAA CCAGCACACTGGA-3′ (forward) and 5′-CTACCAAGGCACAGGGTCAT-3′ (reverse); IL- 12R, 5′-TGCACTGTCAGAGGACCAAG-3′ (forward) and 5′-ATGTCAC CTTGGGCTGTAGG-3′ (reverse); STAT4, 5′-ATCTTCCAATGGGAGCCTCT-3′ (forward) and 5′-GCCCTCGTTTCCTTTACTCC-3′ (reverse); and β-actin, 5′-TACCACAGGCATTGTGATGG-3′ (forward) and 5′- TTTGATGTCACGCACGATTT-3′ (reverse). The cycling condition was 95°C for 30 s, 95°C for 5 s, and 60°C for 34 s. Forty cycles of the profile were run, and the final cooling step was continued for 30 s at 4.0°C. Amplicon size and specificity were confirmed by the melting curve analysis. Relative mRNA expression of the target gene was defined as 2^−ΔΔCt^.

### Western Blot

For Western blot analysis, tumor tissues were homogenized in RIPA buffer in the presence of protease inhibitors. Proteins (40 μg) in the total tissue lysate were separated by sodium dodecyl sulfate polyacrylamide gel electrophoresis (8.0% separation gel, 4.5% spacer gel). After electrotransfer onto nitrocellulose membranes (MSI, Westboro, MA, USA), the membranes were blocked for 1 h at room temperature with nonfat milk. The primary antibodies, goat polyclonal anti-IL-12 antibody (1:1,000, Santa Cruz, CA, USA), rabbit polyclonal anti-IL-12R antibody (1:500, Santa Cruz, CA, USA), rabbit polyclonal anti-pSTAT4 antibody (1:200, Santa Cruz, CA, USA), or mouse monoclonal anti-β-actin (1:2,000, Santa Cruz, CA, USA) was used to probe the blots overnight at 4°C, respectively. Then, membranes were incubated with goat anti-rabbit (1:3,000) or goat anti-mouse (1:3,000) immunoglobulin horseradish peroxidase (Santa Cruz, CA, USA) as a secondary antibody. β-Actin was used as an internal marker for all examined proteins. Protein bands were visualized by the enhanced chemiluminescence (ECL) method (GE Healthcare Life Sciences, Beijing, China). Images were captured by a ChemiDocXRS cooled charge-coupled device camera and analyzed with Quantity One software (Bio-Rad Laboratories, Hercules, CA, USA).

### Statistical Analysis

Data were presented as mean ± SD, and results were analyzed using the SPSS 16.0 software. Statistical significance was determined by one-way analysis of variance, followed by LSD test for multiple comparisons. A value of *P* <0.05 was considered as statistical significant.

## Results

### SSa Inhibited Tumor Growth

The effect of SSa on tumor growth in rats was evaluated in terms of tumor bulk reduction. Tumor volume was largest in the control group and significantly decreased in both therapy groups (*P* < 0.01). The tumor volume reduction rate was 66.29% and 62.84% in the TAM and SSa groups, respectively. Meanwhile, compared with the control group, the tumor weight also significantly decreased in both therapy groups (*P* < 0.05) (**Table 1**). The results indicated that SSa could inhibit breast tumor growth.

**Table 1:** SSa inhibited tumor growth of DMBA-induced breast cancer in rats.

Group	Tumor volume (cm^3^)	Tumor volume reduction rate (%)	Tumor weight (g)
Control	6.21 ± 1.12	–	6.39 ± 3.60
TAM	2.11 ± 0.87**	66.29	1.97 ± 1.26**
SSa	2.31 ± 0.89**	62.84	2.80 ± 1.82*

Data were expressed as mean ± SD (n* = *12). *P < 0.05, **P < 0.01 vs. control group.

### SSa Inhibited Tumor Cell Proliferation

**Figure 1:**
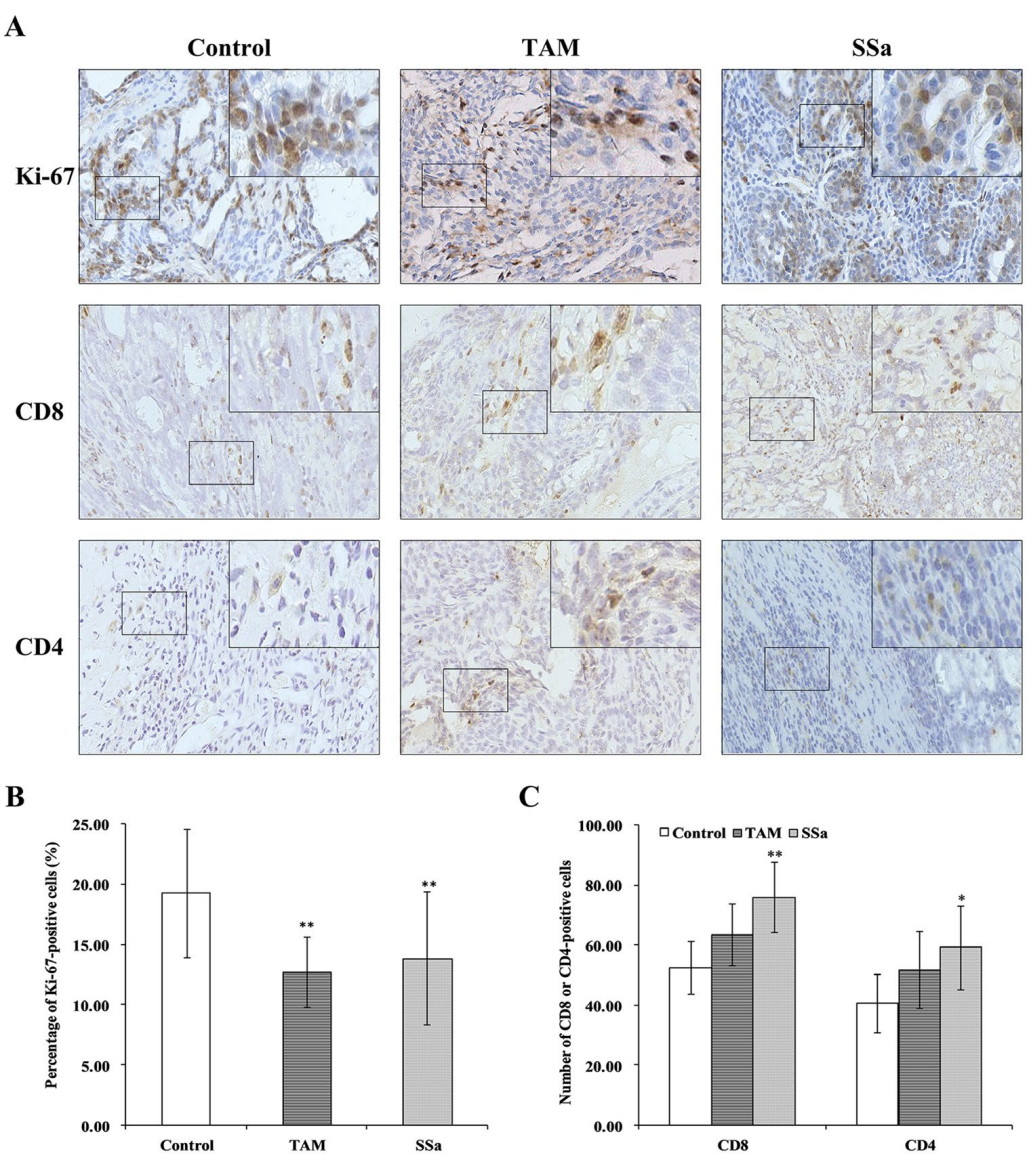
The effects of SSa on tumor cell proliferation and infiltrated T lymphocytes in tumors. **(A)** Immunohistochemistry staining of Ki-67, CD8, and CD4 in rat tumors. **(B)** Percentage of Ki-67-positive cells. **(C)** Number of CD4+ or CD8+ cells. Data were expressed as mean ± SD (*n* = 12). *P < 0.05, **P < 0.01 vs. control group.

The Ki-67 is a proliferative biomarker of tumor cells, and it is associated with poor prognosis in breast cancer patients (Petrelli et al., 2015). The IHC staining of Ki-67 was performed in tumor tissues. In the control group, positive Ki-67 mainly expressed in the nucleus of tumor cells, and the staining was strong. It was weaker in both therapy groups than in the control group (**Figure 1A**). The morphometry results showed that after TAM or SSa treatment, the Ki-67 positive-stained tumor cells were significantly decreased compared with the control group (*P* < 0.01) (**Figure 1B**).

### SSa Regulated Immune Cell Infiltration in Tumors

The IHC stainings of CD8 and CD4 were performed to determine infiltrated CD8+ and CD4+ T cells in tumors.

CD8 positive staining was mainly located in membranes of T cells (**Figure 1A**). The morphometry results showed that the number of CD8 cells infiltrated in tumors was higher in the SSa group than in the control group (*P* < 0.01). There was no significant difference between the control and TAM groups (**Figure 1C**). The CD4-positive staining was mainly located in membranes of the T cells (**Figure 1A**). The morphometry results showed that the number of CD4 cells infiltrated in tumors was higher in the SSa group than in the control group (*P* < 0.05). There was no significant difference between the control and TAM group (**Figure 1C**). The above results indicated that SSa increased infiltration of CD8+ T cells and CD4+ T cells in tumors.

### SSa Promoted Th1/Th2 Shifting Into Th1

**Figure 2:**
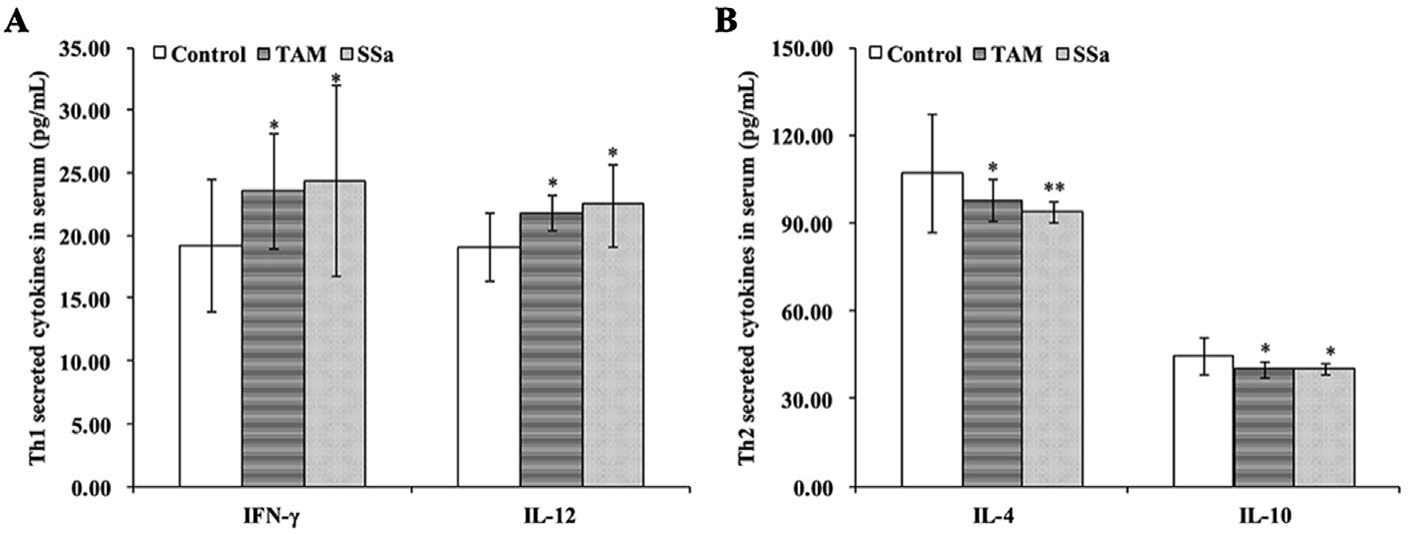
The effects of SSa on cytokines secreted by Th1 or Th2 cells. **(A)** Serum levels of Th1 cytokine IFN-γ and IL-12. **(B)** Serum levels of Th2 cytokine IL-4 and IL-10. Data were expressed as mean ± SD (*n* = 12). *P < 0.05, **P < 0.01 vs. control group.

The cytokines released from Th1 (IFN-γ and IL-12) and Th2 (IL-4 and IL-10) were detected to confirm effects of SSa on antitumor immune response of T cells. The results showed that compared with the control group, TAM and SSa treatment significantly promoted IFN-γ and IL-12 secretion (**Figure 2A**), while it inhibited IL-4 and IL-10 secretion (**Figure 2B**). These results indicated that SSa could induce Th1/Th2 shifting into Th1 cell response.

### SSa Activated the IL-12/STAT4 Pathway

The activation of the IL-12/STAT4 pathway mediated differentiation of Th1 cells. To identify the role of SSa on the IL-12/STAT4 pathway, the mRNA levels of IL-12, IL-12R, and STAT4 were accessed with real-time quantitative RT-PCR, and the protein amounts or IHC expressions of IL-12, IL-12R, and pSTAT4 were detected by Western blot or IHC staining.

**Figure 3:**
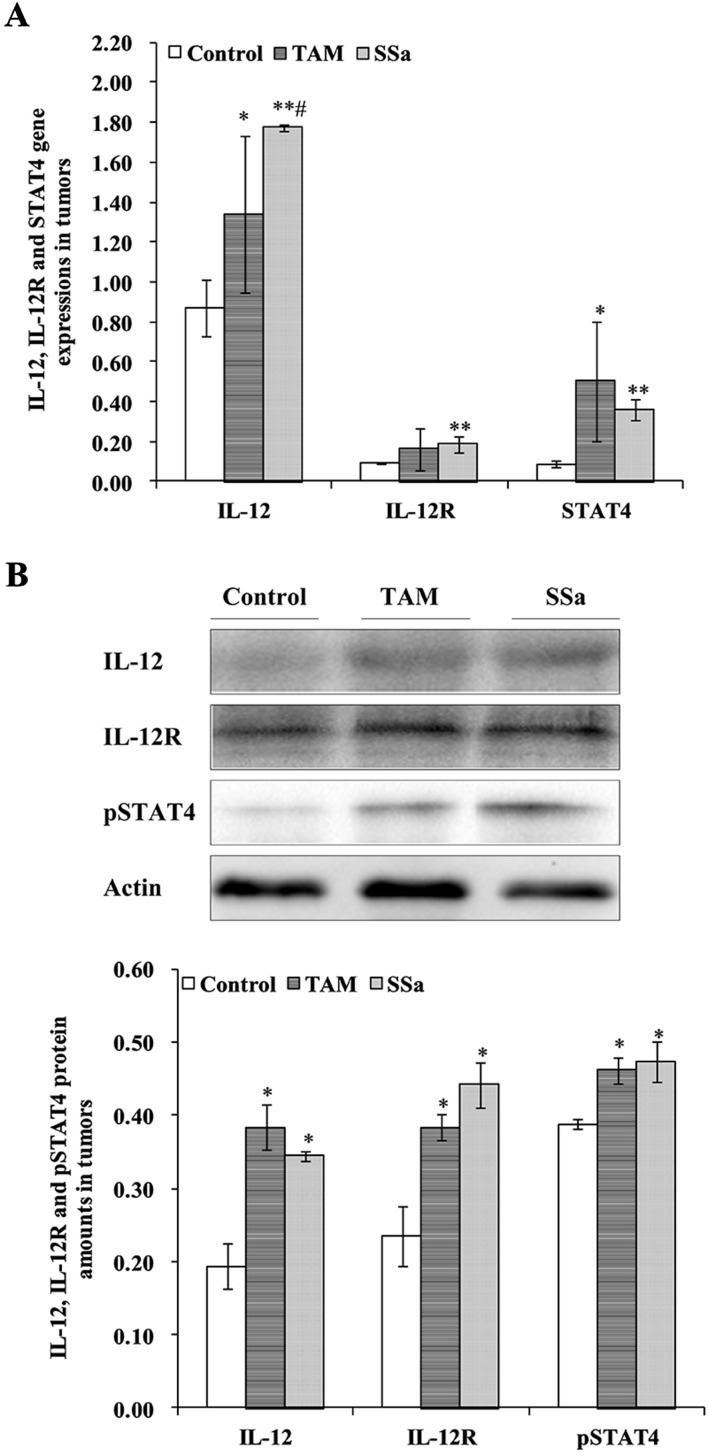
The effects of SSa on gene expression and protein amounts of IL-12, IL-12R, and STAT4. **(A)** Gene expressions of IL-12, IL-12R, and STAT4 in tumors. **(B)** Protein amounts of IL-12, IL-12R, and pSTAT4 in tumors. Data were expressed as mean ± SD (*n* = 12). *P < 0.05, **P < 0.01 vs. control group. ^#^ *P* < 0.05 vs. TAM group.

**Figure 4:**
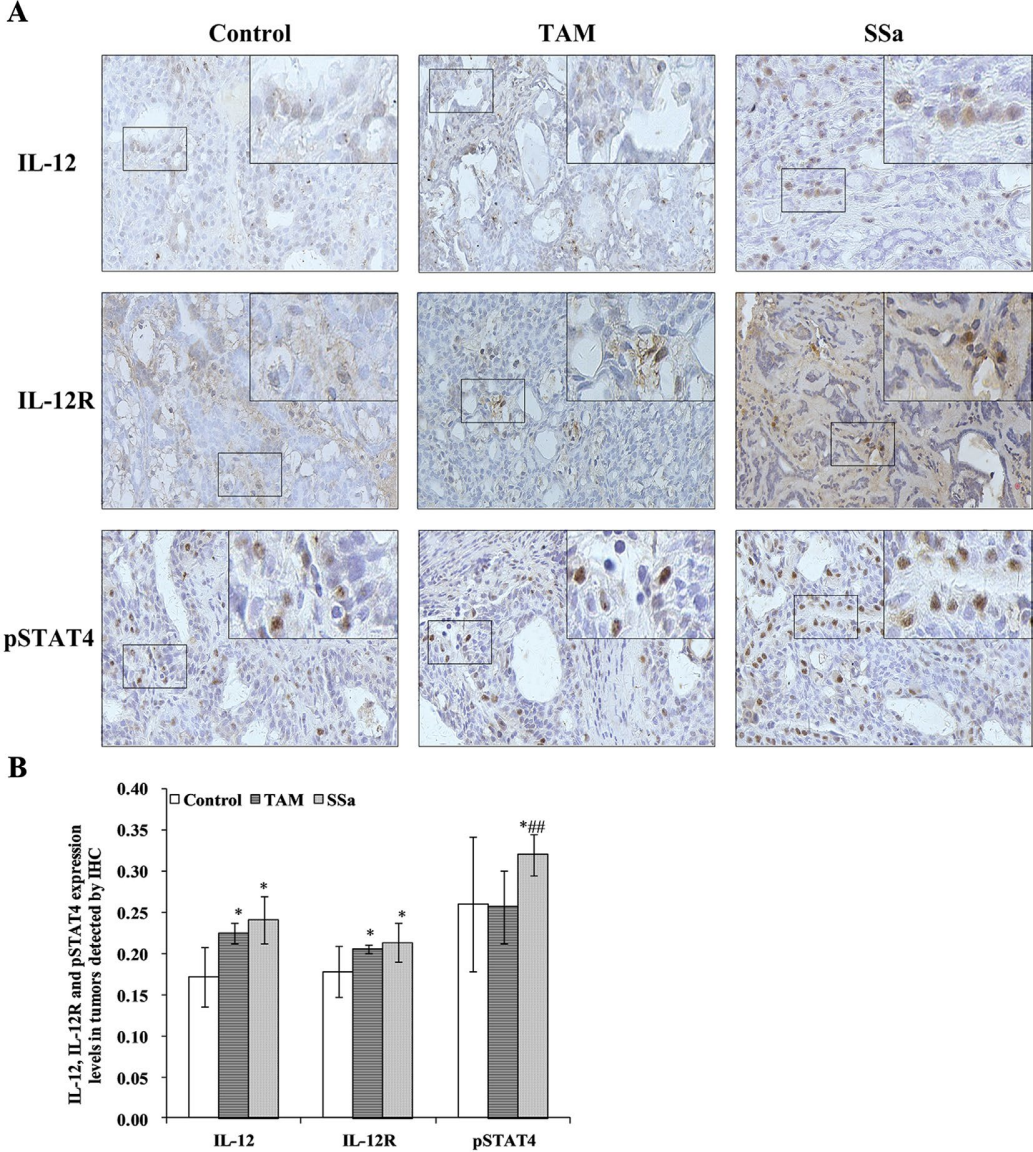
The effects of SSa on ***in situ*** expressions of IL-12, IL-12R, and pSTAT4 in tumors. **(A)** Immunohistochemistry staining of IL-12, IL-12R, and pSTAT4 in rat tumors. **(B)** Morphometric analysis of IL-12, IL-12R, and pSTAT4 expression levels in rat tumors. Data were expressed as mean ± SD (*n* = 12). *P < 0.05 vs. control group. ^##^ *P* < 0.01 vs. TAM group.

IL-12 mRNA expression in the SSa group was significantly upregulated compared with that in the control (*P* < 0.01) and TAM groups (*P* < 0.05), respectively (**Figure 3A**). The IL-12 protein amount significantly increased in the TAM (*P* < 0.05) and SSa (*P* < 0.05) groups compared with that in the control group, respectively (**Figure 3B**). Positive IL-12 staining was located in the cytoplasm of lymphocytes and tumor cells (**Figure 4A**). The IL-12 IHC expressions in both therapy groups were higher than that in the control group (*P* < 0.05) (**Figure 4B**).

IL-12R mRNA expression was significantly upregulated in the SSa group compared with that in the control group (*P* < 0.01), while there was no significant difference between the control and TAM group (**Figure 3A**). IL-12R protein amount significantly increased in both therapy groups compared with that in the control group (*P* < 0.05) (**Figure 3B**). Positive IL-12R staining was mainly located in membranes of T lymphocytes (**Figure 4A**). The IL-12R IHC expressions in both therapy groups were higher than that in the control group (*P* < 0.05) (**Figure 4B**).

The STAT4 mRNA expression was significantly upregulated in TAM group (*P* < 0.05) and SSa group (*P* < 0.01) compared with that in the control group (**Figure 3A**). The pSTAT4 protein amount significantly increased in both therapy groups compared with that in the control group (*P* < 0.05) (**Figure 3B**). Positive pSTAT4 staining was located in the nucleus of lymphocytes and tumor cells (**Figure 4A**). The pSTAT4 IHC expressions in the SSa groups were higher than that in the control group (*P* < 0.05), while there was no significant difference between the control and TAM group (**Figure 4B**).

The above results indicated that SSa could activate the IL-12/STAT4 pathway.

## Discussion

Lymphocytes play important roles in antitumor immunity (Mohme et al., 2017). Tumor-infiltrating T cells are correlated with improved clinical outcome and survival of breast cancer patients (Criscitiello et al., 2016; de Melo Gagliato et al., 2017). The tumor microenvironment includes a complex network of T cell subpopulations. Among the T cell subgroups, Th1/Th2 balance is involved in the immune response in cancer (Kiyomi et al., 2015). The shifting from Th1 to Th2 response promotes breast cancer progression (Gu-Trantien et al., 2013). SSa, one of a triterpene saponins isolated from the root of *Bupleurum falactum*, exerts antitumor and anti-inflammatory activity and immune regulatory function (Motoo and Sawabu, 1994; Chen et al., 2003; Zhu et al., 2013). It is reported that SSa could regulate T cell response (Sun et al., 2009). Thus, in this study, we investigated the effects of SSa on breast cancer and explored its underlying mechanisms on Th1/Th2 balance.

The DMBA-induced mammary carcinoma in rats was used to evaluate the effects of SSa. DMBA leads to mammary epithelial carcinogenesis and has immunotoxicity that impairs antitumor immunity (Miyata et al., 2001). TAM is an anti-estrogen that is recognized as the first-line endocrine therapy for estrogen receptor-positive breast cancer. It was used as a pharmacodynamic positive control for evaluating the antitumor effect of SSa in this study. We found SSa significantly inhibited tumor growth, and the reduction in tumor size was 62.84% compared to 66.29% in TAM-treated rats. The Ki-67 is a proliferative marker, and it has been used as a discriminant of more aggressive malignant phenotypes in early breast cancer (Petrelli et al., 2015). In SSa-treated animals, the percentage of Ki-67-positive cells significantly decreased. The above results demonstrated antitumor activities of SSa. To observe the effects of SSa on T cells in tumor microenvironments, the different subsets of TILs in tumors were detected. The results showed that SSa significantly increased CD8+ and CD4+ T cell infiltration in tumors. Moreover, the cytokines secreted by Th1 or Th2 cells were detected to demonstrate the actions of SSa on Th1/Th2 balance. It was found that SSa significantly increased Th1-released IFN-γ and IL-12 levels, while decreased Th2-released IL-4 and IL-10 levels. It indicated that SSa shifted the Th1/Th2 balance toward Th1 response. Furthermore, the differentiation of Th1 cells from naive CD4+ T cells is associated with activation of the IL-12/STAT4 pathway (Persky et al., 2005). In order to explore the underlying mechanism of SSa on Th1/Th2 balance, the related molecules of this pathway were detected. Our results showed that SSa upregulated the gene expression of IL-12, IL-12R, and STAT4 and increased protein amounts and *in situ* expressions of IL-12, IL-12R, and pSTAT4 in tumor tissues.

Antitumor immunity is a complex process including innate immunity and adaptive immunity (Gajewski et al., 2013). The adaptive immune responses consist of humoral immunity and cellular immunity (Binnewies et al., 2018). CD8+ T cells and CD4+ T cells play key roles in cellular immunity (Agahozo et al., 2018).

CD8+ T cells exerts tumor-killing activity by interacting with tumor antigens, resulting in direct or indirect cell lysis through releasing perforin, granzyme, and cytokines. The higher level of CD8+ T cells was significantly correlated with breast cancer-specific survival (Mahmoud et al., 2011). In this study, SSa significantly increased CD8+ T cell infiltration in tumors, which could enhance anticancer activities. In addition, the effects of SSa on tumor cell proliferation may be associated with enhancing function of CD8+ T cells.

The naive CD4+ T cells differentiate into Th1 and Th2 cells under different cytokine stimulation. Th1 cells produce IFN-γ and IL-12, which exert anticancer activity. Th2 cells mediate the humoral immune and produce IL-4 and IL-10. In contrast to the Th1 response, the Th2 response can actually promote cancer procession. IL-4 secreted by Th2 cells inhibits the secretion of IFN-γ (Narsale et al., 2018). IL-10 has been identified to dampen Th1 cell responses. Endogenous IL-10 could impair the proliferation of Th1 cells and IFN-γ production (Mosmann and Moore, 1991). Our results showed that SSa increased Th1-secreted cytokines, while it inhibited Th2-secreted cytokines. It indicated that SSa could modulate Th1/Th2 shifting to Th1 cytokine responses.

**Figure 5:**
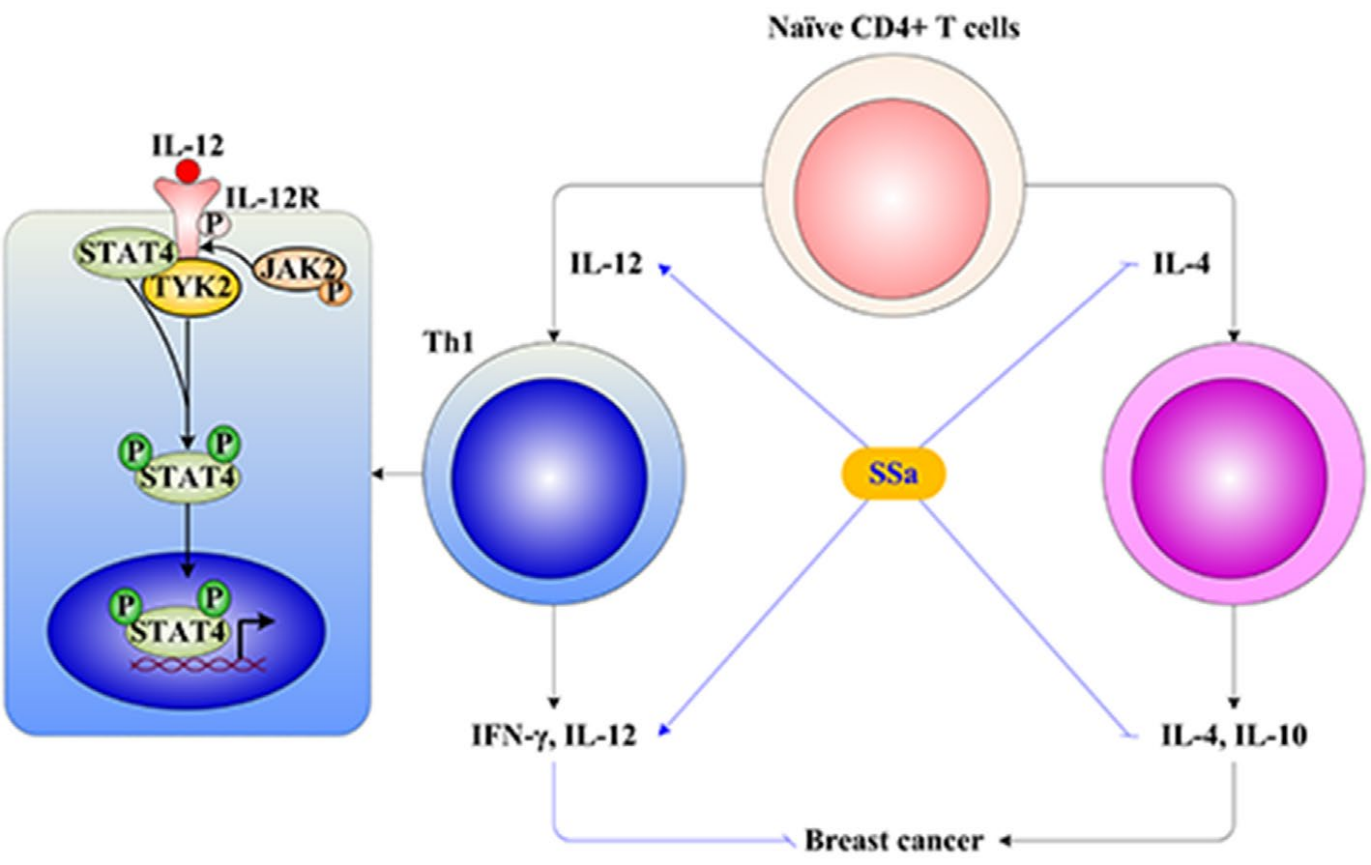
The underlying mechanism of SSa on T cell-mediated immune surveillance in breast cancer. (1) The naive CD4+ T cells differentiate into Th1 or Th2 under IL-12 or IL-4 stimulation, respectively. Th1 produces IFN-γ and IL-12, which exerts anti-breast cancer activity. Th2 produces IL-4 and IL-10, which exerts promoting breast cancer activity. (2) The Th1 differentiation is induced by activation of the IL-12/STAT4 signaling pathway. The IL-12 binds to IL-12R in the membrane of T cells, inducing tyrosine phosphorylation of janus kinase 2 (Jak2) and tyrosine kinase 2 (Tyk2), which in turn phosphorylate IL-12R, providing docking sites for the transcription factor signal transducers and activators of transcription 4 (STAT4). Receptor-bound STAT4 is phosphorylated, which will then dimerize with another STAT4 molecule. STAT4 homodimers translocate to the nucleus and promote IFN-γ gene transcription. (3) SSa increased Th1 released IL-12 and IFN-γ levels, decreased Th2 released IL-4 and IL-10 levels, and promoted Th1/Th2 shifting into Th1 immune response. Furthermore, SSa could upregulate IL-12, IL-12R, and STAT4 expression, activate the IL-12/STAT4 pathway to induce Th1 differentiation, and enhance T cell-mediated immune surveillance of breast cancer.

IL-12 drives the development of Th1 cells *via* activation of STAT4. IL-12 binds its receptor IL-12R in membranes of T cells, inducing tyrosine phosphorylation of janus kinase 2 (Jak2) and tyrosine kinase 2 (Tyk2), which in turn phosphorylates IL-12R, providing docking sites for the transcription factor STAT4. Receptor-bound STAT4 is phosphorylated, which will then dimerize with another STAT4 molecule. STAT4 homodimers translocate to the nucleus and promote IFN-γ gene transcription (Morinobu et al., 2002). In this study, it was found that SSa activated the IL-12/STAT4 pathway through increasing IL-12, IL-12R, and STAT4 gene expressions and elevating IL-12, IL-12R, and pSTAT4 protein levels (**Figure 5**).

## Conclusions

In conclusion, this study demonstrated that SSa inhibits breast cancer. These effects are partially associated with increasing CD8+ T cells and CD4+ T cell infiltration in the tumor microenvironment and promoted Th1/Th2 balance toward Th1 response. The mechanisms might be partially associated with activation of the IL-12/STAT4 pathway. It is demonstrated SSa or its derivates could be explored for breast cancer treatment.

## Ethics Statement

All experimental procedures were approved by the Ethics Committee of the Institute of Medicinal Plant Development, CAMS, and PUMC.

## Author Contributions

DC and YW designed the experiments. XZ, JL, SG, and CC performed the experiments and collected the data. SL, XW, XF, and DC analyzed and interpreted the data. XZ and DC wrote the article. All authors reviewed and approved the manuscript.

## Funding

This work was supported by the National Natural Science Foundation of China (81541082 and 81673674), the Beijing Municipal Natural Science Foundation (7132150), and the Education Ministry Science Foundation of the People’s Republic of China (108019).

## Conflict of Interest Statement

The authors declare that the research was conducted in the absence of any commercial or financial relationships that could be construed as a potential conflict of interest.
